# Microbiota-directed therapies for atopic dermatitis: a three-tier framework for inflammation control, immune modulation, and microbiome restoration

**DOI:** 10.3389/fimmu.2026.1873455

**Published:** 2026-07-15

**Authors:** Yi Zhao, Quan Gao, Guixiu Li, Qiongyan Zhou, Feng Yang, Xiaoxia Zhu

**Affiliations:** 1 Department of Dermatology, The First Affiliated Hospital of Ningbo University, Ningbo, China; 2 Faculty of Medicine, Ningbo University, Ningbo, China

**Keywords:** atopic dermatitis, gut-skin axis, microbial metabolites, microbiota-directed therapy, skin microbiota

## Abstract

The pathogenesis of atopic dermatitis (AD) involves cutaneous barrier dysfunction, immune dysregulation, and microbiota imbalance. Although microbiota-directed therapeutic approaches have garnered increasing attention, current evidence is heterogeneous, encompassing probiotics, postbiotics, microbial metabolites, local microbial interventions, antimicrobials and ecological modulation strategies. This narrative review does not aim to provide clinical guidelines, but rather seeks to synthesise existing evidence within a three-tier conceptual framework. This framework classifies interventions into three categories: those primarily addressing local inflammation and barrier-associated microbiota dysbiosis; those exerting systemic immunomodulation via gut-derived microbial signals; and those modulating the skin microbiota ecology. It should be noted that human clinical data are still limited and heterogeneous, and much mechanistic insight is derived from preclinical research. By clarifying mechanistic layers and evidentiary gaps, this framework may facilitate future research to evaluate the sequencing or combination of anti-inflammatory therapy, barrier restoration, and microbiota ecological modulation.

## 1 Method

This narrative review was conducted by searching PubMed, Web of Science, and Google Scholar for literature using combinations of terms including ‘atopic dermatitis,’ ‘microbiome,’ ‘microbiota,’ ‘probiotics,’ ‘postbiotics,’ ‘faecal microbiota transplantation,’ ‘skin microbiome,’ ‘Staphylococcus aureus,’ ‘short-chain fatty acids,’ and ‘gut–skin axis.’ Priority was given to human studies, randomised controlled trials, and mechanistic studies with direct relevance to atopic dermatitis, as well as recent comprehensive reviews. Preclinical findings were included when they provided mechanistic insights but were interpreted separately from clinical evidence to avoid overgeneralization.

## 2 Introduction

Atopic dermatitis (AD) is a chronic, relapsing, and heterogeneous inflammatory skin disorder, the pathogenesis of which is associated with local cutaneous immune dysregulation, barrier dysfunction, the pruritus–scratch vicious cycle, and microbiota imbalance (1–3). Studies have demonstrated that the onset of AD is not only related to immune-mediated inflammation, but also involves changes in the skin barrier and cutaneous microbiota. Dysbiosis of the skin microbiota may exacerbate inflammation and barrier impairment, whilst inflammation and barrier disruption can in turn alter the skin microbiota (4–7).

Currently, a variety of microbiota-directed therapies (MDTs) are available, including probiotics, prebiotics, postbiotics, antimicrobial treatments, and faecal microbiota transplantation. These interventions are highly heterogeneous; due to differences in study populations, microbial strains, dosages, treatment durations, outcome measures, and levels of evidence, it is not possible to simply determine the effectiveness or ineffectiveness of these therapies. Thus, a framework is required to organise this heterogeneous body of evidence. To clearly summarise the evidence for various MDTs, we have collated key study designs, strain characteristics, and clinical research in **Tables 1**, **2**.

**Table 1:** A multidimensional synthesis of the MDTs tiered intervention framework: from bacterial strains to strength of evidence and efficacy.

First author	MDT type	Intervention	Key findings	Design	Primary target level	Evidence maturity	Onset vs durability
Kim2024 (8)	Postbiotic	*S. pneumoniae*, *S. infantis*, *S. mitis* (3 strains)	IL-13 ↓, CCL17 ↓, CCL22 ↓	Clinical trial	Local inflammation	Early-stage clinical trials	Efficacy measures showed significant improvement at week 8; long-term efficacy data have not been reported
Qiu2022 (12)	Postbiotic	Propionic acid	*HDAC2/3* ↓, IL-33 ↓	Clinical trial	Local inflammation	Early-stage clinical trials	Effective within 1 week (in animals), effective within 2 weeks (in clinical trials); no data on the duration of efficacy are available
Nguyen2023 (13)	Postbiotic	*L. plantarum* K8	*HBD2/HBD3* ↑, TNF-α ↓, IL-1β ↓, IL-6 ↓, IL-8 ↓	Animal study	Local inflammation	Preclinical research	Rapid onset of action within 1 hour (at the molecular level), 24 hours (*in vitro* anti-inflammatory effects), 24 hours (in animal inflammation models); long-term persistence not mentioned
Kim2020 (21)	Probiotics	Mixed Lactobacillus spp.	IgE↓, IL-4 ↓, IL-13 ↓, Filaggrin restoration, Loricrin restoration	Animal study	Systemic immune tolerance	Preclinical research	Effective within one week (animal models); no data on the duration of efficacy are provided
Kang2021 (24)	Probiotics	5-strain mix (LA, LC, LR, BB, ST)	peripheral Treg differentiation ↑, CTLA-4 ↑, IL-10 ↑, Reduced skin inflammation	Animal study	Systemic immune tolerance	Preclinical research	Not mentioned
Kim2021 (27)	FMT	FMT (faecal microbiota)	IL-12 ↑, IFN-γ ↑, TNF-α ↑, IL-4 ↓, IL-5 ↓, IL-13 ↓	Animal study	Systemic immune tolerance	Preclinical research	Gut microbiota diversity was restored within one week (in an animal model) and remained stable for eight weeks, whilst the therapeutic effect was still evident at week eight
Qi2025 (30)	Synbiotic	2’-FL + *B. bifidum* + *B. longum*	Treg differentiation ↑, Th2/Th17 response ↓	Animal study	Systemic immune tolerance	Preclinical research	Not mentioned
Zhou2022 (32)	Probiotics	*L. reuteri* FN041	IgE ↓, IL-4 ↓, IL-33 ↓, TSLP ↓	Animal study	Systemic immune tolerance	Preclinical research	Not mentioned
Fang2022 (50)	Probiotics	*Bifidobacterium longum*	TSLP ↓, IL-4 ↓, IL-5 ↓, AHR activity ↑	(Animal + Clinical trial)	Systemic immune tolerance	Early-stage clinical trials	Significant improvement in symptoms was observed after 8 weeks (clinically); no mention was made of long-term effects
Zhao2025 (54)	Probiotics	*B. lactis* BX-BC08	IL-4 ↓, IL-13 ↓, IL-25 ↓, TSLP ↓, IL-22 ↓, *H3K9me3* demethylation ↑, *H3K27me3* demethylation ↑	Animal study	Systemic immune tolerance	Preclinical research	Symptoms improved within three weeks (animal model)
Ito2021 (59)	Probiotics	*S. cohnii*	TSLP ↓, IL-4 ↓, IL-13 ↓, glucocorticoid synthesis ↑, *TNFAIP3* expression ↑, *ATF3* expression ↑	Animal study	Ecological regulation	Preclinical research	Efficacy was observed within one week and persisted for at least three weeks (animal model)
Myles2018 (62)	Topical MT	*R. mucosa*	SCORAD↓, *S. aureus* colonization ↓, corticosteroid requirement ↓	Clinical trial	Ecological regulation	Early-stage clinical trials + Preclinical research	Symptoms improved significantly after 6 weeks in adults and 16 weeks in children; clinical improvement was still maintained in the adult group 4 weeks after the end of the intervention
Nakatsuji2021 (63)	Topical MT	*S. hominis* A9	SCORAD↓, IL-4 ↓, IL-13 ↓, antimicrobial peptides ↑	Clinical trial	Ecological regulation	Early-stage clinical trials	Significant antibacterial effect within one week (clinical), with the effect persisting for four days in non-lesional areas and four hours in lesional areas
Liu2023 (64)	Topical MT	*R. mucosa*	TEWL ↓, IgE ↓, TNF-α ↓, TSLP ↓, IL-1β ↓, IL-4 ↓, IL-17A ↓	Animal study	Ecological regulation	Preclinical research	Improvement in skin symptoms within 2 weeks (in animals); effects lasting for at least 48 hours

^a^ HDAC, histone deacetylase; HBD, human β-defensin; TSLP, thymic stromal lymphopoietin; Treg, regulatory T cells; LA, *L. acidophilus*; LC, *Lactobacillus casei*; LR, *Lactobacillus reuteri*; BB, *Bifidobacterium bifidum*; ST, *Streptococcus thermophilus*; 2’-FL, 2’-fucosyllactose; SCORAD, SCORing Atopic Dermatitis; TEWL, transepidermal water loss; MT, microbiome transplantation; AHR, aryl hydrocarbon receptor; TNFAIP3, tumor necrosis factor alpha-induced protein 3; ATF3, activating transcription factor 3; FMT, faecal microbiota transplantation.

^b^ Clinical trial registration numbers: KCT0007876, ChiCTR2100043963, NCT03018275, NCT03151148.

**Table 2:** Clinical trials of microbiota-directed therapies in atopic dermatitis/eczema.

Study	Design and participants	MDT type	Intervention	Control	Duration	Endpoint	Key findings	Tier	Safety	Limitations
Kim 2024 (8)	Randomised, double-blind, vehicle-controlled, proof-of-concept; mild-to-moderate AD, 12–70 years, *n=98*	Postbiotic	Strain CX postbiotic cream 1.0%, applied daily; contains S. pneumoniaeBF00257, S. infantisBF00247, S. mitis BF00251	Vehicle cream (without Strain CX)	8 weeks treatment; safety to week 24	Primary: IGA 0/1 with ≥1-point improvement at week 8; Secondary: EASI-25/50, skin hydration, TEWL, pruritus VAS, barrier proteins, serum markers	Significant improvement at week 8: IGA response 41.5% vs 12.1% (*P=0.005*); EASI-25 46.2% vs 9.1% (*P<0.001*); improvements in hydration, TEWL, itch, barrier proteins, cytokines.	I	No significant safety issues reported	No microbiome analysis; limited to mild-to-moderate AD; limited biomarker evaluation
Qiu 2022 (12)	Proof-of-concept trial; mild-to-moderate AD, n=11	Microbial metabolite	Propionate 0.05% cream, applied twice daily to lesional skin	Vehicle cream on contralateral site	2 weeks	Regional SCORAD, TEWL, skin hydration, subjective itch	Propionate side showed improved SCORAD, TEWL, hydration, and itch at week 2 vs vehicle	I	No significant safety issues reported	Very small sample; only 2-week follow-up; proof-of-concept only; excluded >45 years
Cabana 2017 (34)	Randomised, double-blind, parallel (TIPS); high-risk infants, n=184	Probiotic	*L. rhamnosus* GG 10^10 CFU + inulin 225mg, daily for 6 months	Inulin 325mg (placebo)	6 months; median follow-up 4.6 years	Primary: 2-year eczema incidence; Secondary: 5-year asthma/allergic rhinitis incidence	Eczema 2-year incidence: 28.7% vs 30.9% (HR 0.95, P = 0.83); no significant difference	II	No major adverse events reported	Lower-than-expected AD incidence; possible insufficient power; long breastfeeding may mask effect
Barbarot 2025 (35)	Multicentre, randomised, double-blind, placebo-controlled; high-risk infants; n=376	Prebiotic	GOS/inulin mixture (9:1), 11.8g/day from week 20 of gestation to delivery	Maltodextrin powder, 12.4g/day (placebo)	From week 20 of gestation to delivery; follow-up to 1 year	Primary: AD prevalence at 1 year; Secondary: 6-month AD (ISAAC), SCORAD, POEM, FDLQI, TEWL, allergen sensitisation, food allergy, prebiotic tolerance	No significant difference in AD prevalence (20.2% vs 20.2%, OR=1.01, P = 0.97); no significant difference in secondary endpoints	II	Similar rate of digestive adverse events in both groups; no serious adverse events	Lower-than-expected AD prevalence; intervention only during pregnancy; limited microbiome validation
Anania 2025 (36)	Single-centre, randomised, double-blind, placebo-controlled; atopic family history; n=74 (41 completed)	Probiotic	*B. bifidum*PRL2010 10^9 CFU/day; mothers from week 36 gestation to 3 months postpartum, infants 3–6 months	Placebo: maltodextrin	Mothers: week 36 gestation to 3 months postpartum; infants: 3–6 months; follow-up to 12 months	Primary: AD incidence at 12 months; Secondary: SCORAD, xerosis, pruritus, cradle cap, SPT	AD incidence lower in probiotic group at all timepoints but not significant; better improvement in severe cases	II	No severe or mild adverse events reported; confirmed safety in pregnancy and lactation	Very small sample; high dropout; single centre; no TEWL, IgE, cytokine data; did not distinguish IgE-AD subtypes
Dotterud 2010 (37)	Randomised, double-blind, placebo-controlled; unselected pregnant women; n=415	Probiotic (combination)	Biola® milk: *L. rhamnosus* GG, *B. animalis* Bb-12, *L. acidophilus*La-5, 250mL/day from week 36 to 3 months postpartum	Placebo: heat-treated, sterile fermented milk	Week 36 gestation to 3 months postpartum; follow-up to 2 years	Primary: 2-year cumulative AD, asthma, allergic rhinoconjunctivitis, atopic sensitisation; Secondary: NESS, IgE-AD, SPT, specific IgE	Significant reduction in cumulative AD (OR=0.51, P=0.013, NNT=8), greater effect in non-IgE AD, no effect on asthma/rhinitis	II	No adverse events reported	High dropout; only maternal intervention; no microbiome validation; follow-up only to age 2
Fang 2022 (50)	Randomised, placebo-controlled; AD patients, n=87 (44 intervention, 43 placebo), mean age 47–50	Probiotic	*B. longum*CCFM1029 10^9 CFU/d for 8 weeks	Placebo: maltodextrin 2g/d	8 weeks; visits at 0, 4, 8 weeks	Primary: SCORAD, DLQI; Secondary: serum IgE, IL-4, faecal/serum tryptophan metabolites, gut microbiota, PICRUSt	Significant reduction in SCORAD, DLQI, IgE; increased I3C; I3C negatively correlated with SCORAD, DLQI; altered β-diversity	II	No adverse events; no significant changes in serum biochemistry or urine	No long-term follow-up; only one B. longum strain effective; post hoc response analysis; no metagenomic validation
Myles 2018 (62)	Open-label phase I/II safety and activity (BACTERiAD I/II); adults n=10, children n=5; total n=15	Live biotherapeutic	*R. mucosa* (3 strains), escalating dose, topical; adults: twice/week ×6 weeks, children: twice/week ×12 weeks, then every other day for 4 weeks	No control; standard care maintained	Adults: 6 weeks + 4-week washout; children: 16 weeks + 10–12 mo follow-up	Primary: safety; Activity: SCORAD-50; Secondary: local/total SCORAD, itch, QOL, S. aureus/CoNS ratio, TEWL, IgE, steroid use	No adverse events or complications; 10/15 achieved SCORAD-50 (P=0.016); S. aureus/CNS ratio reduced; hand treatment ineffective	III	No adverse events or complications; no lab changes in children; no toxicity in animal model	Open-label; no control; small sample; short adult treatment; no microbiome genomics; hand treatment ineffective; response linked to family history
Nakatsuji, 2021 (63)	Randomised, double-blind, vehicle-controlled, multicentre phase I; moderate-to-severe adult AD (volar forearm); n=54	Live biotherapeutic	*S. hominis* A9 (ShA9), 1×10^9 CFU/g, applied twice daily for 7 days	Vehicle (Cetaphil/glycerol/saline); stable regimens allowed	7 days; follow-up to 96h post-treatment (days 8, 9, 11); phone ~30 days	Primary: safety; Secondary: local EASI, SCORAD, itch VAS; S. aureus/CoNS, ShA9 DNA/mRNA, 16S rRNA,	Fewer adverse events (55.6% vs 83.3%, P=0.044); significant reduction in S. aureus; subgroup with S. aureus sensitivity: improved EASI/SCORAD	III	No serious adverse events; mild eczema, pain, swelling; 1 moderate boil (self-resolving); no systemic infection	Small sample; only 1-week treatment + short follow-up; only S. aureus-positive adults included; no long-term safety data

^a^ AD, Atopic dermatitis; MDT, Microbiota-directed therapy; IGA, Investigator’s Global Assessment; EASI, Eczema Area and Severity Index; SCORAD, SCORing Atopic Dermatitis; DLQI, Dermatology Life Quality Index; TEWL, Transepidermal Water Loss; VAS, Visual Analogue Scale; QOL, Quality of life; CoNS, Coagulase-negative staphylococci; SPT, Skin prick test. NESS, Nottingham Eczema Severity Score; IgE, Immunoglobulin E; IL-4, IL-13, Interleukin-4, Interleukin-13; sIL-2R, Soluble Interleukin-2 Receptor; CCL17, CCL22, Chemokine (C-C motif) ligand 17/22; I3C, Indole-3-carbinol; AIP, Autoinducing peptide; HR, Hazard ratio; OR, Odds ratio; CI, Confidence interval; NNT, Number needed to treat; PICRUSt, Phylogenetic Investigation of Communities by Reconstruction of Unobserved States.

In this review, we classify various MDTs according to their mechanisms of intervention: local inflammation control, systemic immunomodulation via the gut–skin axis, and modulation of the cutaneous microbiota. These three levels are not mutually exclusive therapeutic categories, but represent the primary mechanistic orientations, with different interventions potentially involving more than one level. MDTs are defined as interventions that specifically target the composition, function, metabolites, or host interaction capacity of the skin or gut microbiota.

This narrative review does not aim to provide clinical treatment guidelines, nor to offer an exhaustive inventory of current AD therapies. Instead, it synthesises the existing evidence on microbiota interventions and organises it into a three-tier conceptual framework: local inflammation control, systemic immunomodulation, and microbiota ecological modulation. This framework aims to elucidate mechanistic differences, identify translational research gaps, and support the design of future combination or sequential therapeutic strategies.

## 3 Regulation of local inflammation

Clinically, topical formulations are considered the first-line local intervention for the skin. For the topical treatment of AD, the following outlines how topical formulations may synergistically intervene through three distinct strategies: immunomodulation (such as via microbiota and their metabolites), rapid anti-inflammatory action (for example, through small-molecule metabolites), anti-infective effects, and barrier restoration (such as by antimicrobial peptide modulators). Kim et al. developed a mixture comprising inactivated cells and metabolites from three skin-derived Streptococcus strains, applied directly to keratinocytes and the skin microenvironment, specifically inhibiting Th2-type inflammatory effectors including interleukin-13 (IL-13), chemokine ligand 17 (CCL17), and CCL22, thereby achieving local immunomodulation. In patients with AD, after eight weeks, 41.5% exhibited near-complete clearance of lesions, and the intervention group showed an average reduction in transepidermal water loss (TEWL) of 3.95 *g/h/m²* (8). Due to the absence of pathway inhibitor validation, the blockade of Th2 cell activation can only be inferred from changes in biomarkers, which may be related to the suppression of direct dendritic cell–T cell interactions by Streptococcus fermentation products.

Although postbiotic emollients have demonstrated anti-inflammatory effects when applied locally to the skin, their efficacy only becomes fully apparent after eight weeks, and these emollients typically contain macromolecules with limited skin penetration. Propionate, a microbiota-derived metabolite, can be formulated into a highly permeable propionate cream, addressing the need for rapid-response therapy. Previous studies have shown that *Staphylococcus aureus (S. aureus)* induces the production of IL-33, which acts as a pro-inflammatory mediator, thereby exacerbating the inflammatory response in AD (9–11). Mechanistically, propionate rapidly inhibits histone deacetylase 2/3 (*HDAC2/3*) in keratinocytes, enhances Aryl hydrocarbon receptor (AhR) expression, and induces AhR nuclear translocation, suppressing IL-33 transcription. *In vitro* studies demonstrated that propionate reversed the increase in IL-33 expression in keratinocytes after 24 hours. Importantly, in patients with mild-to-moderate AD (*n = 11*), after two weeks, the regional SCORAD (SCORing Atopic Dermatitis) score was significantly reduced, with marked improvements in subjective pruritus and TEWL, showing a notably faster therapeutic response than postbiotic emollients (12).

It is noteworthy that, whilst propionate inhibits IL-33 production, it does not possess direct bactericidal activity. For cases where *S. aureus* colonisation may lead to skin infection and barrier disruption, MSF (fermentation extract of *Lactobacillus plantarum* K8) can synergistically target barrier defects by enhancing skin barrier function and reducing inflammation-driving factors. *In vitro* studies demonstrated that MSF rapidly induced the expression of human β-defensin-2/3 (*HBD2/HBD3*), with sustained high expression over 48 hours, thereby strengthening barrier protection. Additionally, in pre-treated *in vitro* inflammation models, MSF exerted negative feedback inhibition on *p38/NF-κB* phosphorylation, with significant reductions in tumour necrosis factor-α (TNF-α), IL-1β, IL-6, and IL-8 inflammatory mediators (13). MSF addresses gaps in antimicrobial defence and barrier restoration. However, the effect of MSF on *HBD2/3* expression in human skin tissues has not been validated. The protective and antimicrobial roles of HBD-2 in the AD barrier have been previously reported in the literature (14, 15).

This tier of intervention is limited by its short duration, localised effect, and inability to achieve long-term immune regulation. Despite observed therapeutic efficacy, evidence is largely derived from short-term studies, and the sustained reduction in pathogenic burden and durability of barrier restoration require further confirmation. Within this framework, these three topical therapies may be suited as transitional interventions for the acute, subacute, and maintenance phases in patients with mild-to-moderate AD, rather than as independent long-term strategies.

## 4 Systemic immune modulation

In the following text, this category includes interventions whose primary mechanism of action is not directed at the affected skin, but which may influence atopic dermatitis via intestinal metabolites, systemic immune modulation, and immune regulatory circuits. The current evidence is derived primarily from preclinical studies, whilst human data are both limited and heterogeneous.

### 4.1 Gut-derived metabolites: SCFAs and immune regulation

Short-chain fatty acids (SCFAs) exert an influence on AD via the gut–skin axis, with studies demonstrating that faecal SCFA concentrations and the abundance of SCFA-producing gut microbiota are closely associated with AD (16, 17). Moreover, SCFAs have been shown to promote the differentiation of regulatory T cells (Treg) and enhance the expression of cutaneous barrier proteins, highlighting the importance of SCFAs in both immunomodulation and barrier protection (18, 19). Simultaneously, in other allergic diseases such as chronic spontaneous urticaria, a reduction in the principal SCFA-producing microbiota has also been observed (20).

In murine models, oral administration of *Lactobacillus* spp. for one week resulted in decreased markers of intestinal inflammation (IL-6, IL-1β, S100A8/A9), accompanied by reductions in IgE and Th2-type cytokines (IL-4, IL-13), alongside a trend towards improved epidermal barrier function. This preclinical study suggests that the alleviation of gut inflammation may be involved in the Lactobacillus-mediated systemic immunomodulation and improvement of skin barrier function; however, the causal relationship along the ‘inflammation–immunity–barrier’ axis and the applicability in human populations remain to be further established (21). Mechanistically, Kim, building on findings by Maslowski in a colitis model, hypothesised that oral probiotics modulate gut microbiota composition, increase SCFA production, and subsequently activate GPR43 to exert anti-inflammatory effects (21, 22). The study by Maslowski et al. demonstrated that the SCFA–GPR43 signalling axis plays a pivotal role in the regulation of intestinal inflammation, providing a mechanistic basis for immunomodulation by probiotics through their metabolites. Previous studies have confirmed, through multilayered experiments, that butyrate induces interleukin-22 (IL-22) production via GPR41 (rather than GPR43 or GPR109A), yet *in vitro* experiments have shown that SCFAs (acetate, propionate, and butyrate) can all enhance the generation of CD4^+^ T cells and IL-22 (23).

On this basis, another genetic model study found that prophylactic administration of the composite probiotic preparation IRT5 suppressed contact hypersensitivity (CHS). The specific involvement of GPR43 was further confirmed by the markedly diminished or absent protective effect in GPR43 knockout mice, as evidenced by increased ear thickness, failed Treg induction, and loss of IFN-γ suppression (24). These findings suggest that the SCFA–GPR43 axis may represent a key mechanism by which probiotics modulate T cell responses and ameliorate cutaneous inflammation. Specifically, SCFAs may promote the expansion of peripherally induced Treg cells (pTreg) via binding to GPR43 on T cell surfaces, thus participating in this immunoregulatory process. It should be noted that current evidence for GPR43-dependent mechanisms is predominantly derived from CHS models; although IRT5 intervention also demonstrated cutaneous anti-inflammatory effects in AD models (24), it remains to be clarified whether these effects are similarly dependent on the SCFA–GPR43 axis.

Although *GPR43* knockout abolished the therapeutic effect of IRT5, single butyrate-producing strains did not exhibit efficacy, and given the complexity of multi-strain formulations, the possibility that other strains exert auxiliary effects via GPR43-independent pathways cannot be completely excluded. Future studies employing conditional knockout models for GPR43 are required to further validate this pathway.

### 4.2 FMT: promising but preclinical

Preclinical studies suggest that oral administration of probiotic preparations capable of elevating intestinal SCFA levels may help alleviate cutaneous inflammation. However, intestinal colonisation by these probiotics may be relatively transient, their influence on local microbiota composition and microbial metabolite concentrations may be limited, and intervention with a single metabolite (such as butyrate) may not consistently yield stable or significant therapeutic effects (24–26).

In murine models of AD, faecal microbiota transplantation (FMT) has been employed to transfer gut microbiota from healthy donors. Following FMT, recipient mice exhibited increased intestinal SCFA levels, accompanied by alterations in Th1/Th2-associated cytokine profiles, including upregulation of IL-12 and IFN-γ, as well as downregulation of IL-4, IL-5, and IL-13, suggesting that FMT may participate in modulating the immune imbalance associated with AD-like inflammation in this model (27). However, it should be noted that both IL-10 and IL-1β decreased post-FMT, yet IL-10 levels remained significantly higher than those in the donor group (*p = 0.0007*), indicating that immune status had not fully reverted to that of healthy donors. Furthermore, improvements in Th1/Th2-related indices were not accompanied by complete normalisation of the Treg axis, suggesting that the impact of FMT on immune tolerance-related pathways may remain insufficient in this animal model.

In AD animal models, FMT has demonstrated multi-pathway regulatory potential, evidenced by elevated intestinal SCFA levels, upregulation of programmed death ligand 1 (PD-L1), and a shift in Th1/Th2-related cytokines towards inflammation resolution (27). Following FMT, gut microbiota diversity recovered within one week and was maintained for up to eight weeks; concurrently, elevated SCFA levels, upregulated PD-L1 (CD274) expression, and a Th1/Th2 balance favouring inflammation alleviation were observed at eight weeks post-intervention (27).

At present, research into FMT for the treatment of atopic dermatitis relies primarily on data from animal models, which suggest that it has potential anti-inflammatory effects in terms of modulating the composition of the gut microbiota and immune responses. However, these preclinical findings have not yet been sufficiently validated in terms of clinical efficacy and safety. In clinical practice, particular attention must be paid to potential safety risks associated with antibiotic pre-treatment, such as secondary infections, antibiotic resistance and microbial imbalance (28). Therefore, future clinical studies should not only systematically evaluate the therapeutic efficacy of FMT but also strengthen safety monitoring to ensure that treatment risks are minimised and patient interests are safeguarded.

### 4.3 Early-life microbial programming and AD risk

The aforementioned studies suggest that a sole reliance on short-term immunomodulation may be insufficient to fully explain changes in immune homeostasis over the long-term course of AD. Given that existing research suggests that the development of atopic dermatitis may be linked to immune development in early life (29). In this context, whether the early microbiota-metabolite axis plays a role in regulating AD-related immune susceptibility has emerged as a promising area of research. Consequently, a number of animal studies have begun to explore the potential role of the early microbiome and its metabolites in the development of the immune system and in AD-related immune homeostasis.

A study by Qi et al. on cross-feeding with Bifidobacterium and 2’-fucosyllactose (2’-FL) demonstrated that this intervention increases SCFA production in animal models. Furthermore, RNA sequencing of ileal Peyer’s patches (PPs) revealed that pathways related to retinol metabolism were activated. Concurrently, the abundance of tolerance-related cells, such as immature dendritic cells (DCs) and Tregs, within the PPs was also increased (30). Notably, Spearman analysis indicated a negative correlation between SCFA levels and AD inflammatory markers (IL-33, thymic stromal lymphopoietin [TSLP], etc.), suggesting that SCFAs may be involved in the modulation of AD-like inflammation via the gut microbiota. Qi et al. also observed that plasma retinoic acid (RA) levels correlated with ear skin thickening, IL-33, mast cells, TSLP, and final AD scores in the animal model (30).

Population cohort studies have also provided indirect support for the association between early-life microbiota changes and AD risk. The CHILD cohort study (*n = 1115*) showed that antibiotic exposure before one year of age was associated with an increased risk of AD at five years (*OR=2.25, 95% CI 1.55-3.27*), accompanied by a reduction in SCFA-producing microbiota at one year (31). However, this study primarily suggests an association between early antibiotic exposure, changes in SCFA-producing microbiota, and subsequent AD risk, without establishing that SCFAs directly mediate the development of immune tolerance or provide long-term protective effects. Furthermore, although SCFA and RA levels are correlated, direct evidence that SCFAs regulate the activity of retinol metabolism-related transcription factors in PPs is currently lacking. Thus, the role of the PPs–retinol metabolism pathway as a major mechanism by which the gut microbiota ameliorates AD-like inflammation requires further investigation.

In line with this, Zhou et al. reported in animal models that supplementation with *Lactobacillus reuteri* FN041 during late pregnancy and lactation could influence the gut microbiota of offspring through breast milk secretory immunoglobulin A (sIgA)-related pathways, activate the retinol metabolism pathway, upregulate alcohol dehydrogenase (ADH), retinal dehydrogenase (RALDH), and *ALDH1A2* expression, and promote RA production (30, 32). This intervention also reduced Th2-associated cytokines (IL-4) and ear tissue levels of IL-33/TSLP. Compared with continuous intervention during late pregnancy and lactation, supplementation with the strain after weaning had a diminished effect on the suppression of Th2 inflammatory cytokines, suggesting the existence of an early window of greater susceptibility to microbiota interventions. However, this conclusion is mainly based on animal models and cannot yet be directly extrapolated to the prevention or long-term immune remodelling of human AD.

Further research indicates that neonatal skin CD301b+ DC2 cells can generate RA via RALDH2, participating in the differentiation of symbiotic microbiota-reactive Tregs and the establishment of cutaneous tolerance (33). Collectively, these findings suggest that early microbiota, SCFAs, and RA-related pathways may contribute to the regulation of cutaneous immune homeostasis. However, their causal roles, critical time windows, and long-term effects in the pathogenesis and progression of AD require further elucidation.

### 4.4 Probiotics/prebiotics and clinical heterogeneity

Randomised, double-blind trials in individuals at high risk of allergy have shown that supplementation with *Lactobacillus rhamnosus* GG (LGG) for six months after birth did not confer a preventive effect against infantile eczema (34).Similarly, the PREGRALL study, which implemented prebiotic intervention in pregnant women with a history of atopic disease starting from 20 weeks’ gestation, found no statistically significant difference in the prevalence of AD at one year of age between the intervention and placebo groups (20.2% vs 20.2%, *OR = 1.01, P = 0.97*) (35). In another study supplementing mothers with a history of atopic disease and their infants with Bifidobacterium bifidum PRL2010. There was no significant difference in AD incidence (4.17% vs 11.76%*, P = 0.3700*); however, amongst infants who developed severe AD, the probiotic group demonstrated a superior SCORAD improvement rate compared to placebo (66.91% vs 31.50%) (36).

In contrast, another randomised double-blind trial enrolling non-selected pregnant women found that perinatal supplementation (from four weeks before delivery to three months postpartum) with a multi-strain probiotic containing LGG, *Lactobacillus acidophilus* La-5, and *Bifidobacterium animalis* subsp. *lactis* Bb-12 resulted in a lower incidence of AD in offspring at two years of age (*OR = 0.51*) (37). This finding suggests that the efficacy of microbial interventions for AD prevention may be inconsistent across studies, potentially influenced by the characteristics of study populations, intervention protocols, and endpoints.

The differences observed amongst the above clinical trials may be attributable to several factors. Firstly, the timing of intervention may be critical, as postnatal supplementation and perinatal intervention target different developmental windows. Secondly, the effects of different strains or strain combinations may vary (38, 39). Some preclinical studies suggest that not all strains can replicate protective effects, and some may even exhibit divergent immunomodulatory actions depending on timing (24, 32). Thirdly, the strength of effect between single-strain and multi-strain interventions may differ, and whether there is a synergistic effect with combination preparations requires further investigation (24). In addition, individual baseline microbiota composition and host responses may also influence intervention outcomes; existing research indicates that, even when overall intervention effects are observed, those who develop AD post-intervention may possess distinct gut microbiota profiles (40). Lastly, heterogeneity in inclusion criteria, such as whether only individuals at high familial risk of allergy are enrolled, may also contribute to differences in study outcomes.

In summary, current clinical evidence regarding the prevention of AD with probiotics or prebiotics remains inconsistent. Future studies should further optimise research design with respect to timing of intervention, target populations, strain selection, and host stratification.

### 4.5 Molecular recognition-based interventions

Although MDTs have demonstrated some efficacy in animal models and select clinical studies, for patients with established AD or those who have missed the early intervention window, a sole reliance on overall microbiota modulation may be insufficient to achieve stable therapeutic effects. Accordingly, recent research has begun to shift from ‘microbiota composition modulation’ towards the identification of ‘microbiota–host interaction molecules’, with the aim of discovering more controllable and quantifiable therapeutic targets. The AhR is one of the most studied pathways in the regulation of cutaneous inflammation and barrier function. Topical AhR agonists have been shown in clinical trials to improve lesion severity and pruritus in AD (41).

Mechanistic studies indicate that active components of coal tar can activate AhR in keratinocytes. Once activated, AhR regulates Th2-related inflammatory responses via the AhR–nuclear factor erythroid 2-related factor 2 (NRF2)–signal transducer and activator of transcription 6 (STAT6) signalling axis. Furthermore, AhR is involved in the regulation of multiple skin barrier proteins (42). However, some exogenous AhR ligands may pose exposure-related toxicity or safety concerns, limiting their long-term application. Therefore, identifying endogenous or commensal microbiota-derived AhR ligands with greater physiological compatibility has become an important direction in the molecular research of MDTs. Metabolites produced by the gut microbiota can also interact with AhR (43, 44). Thus, the search for endogenous or symbiont-derived AhR ligands with improved physiological compatibility is a key strategy for enhancing the safety and precision of molecular therapies.

Indole-3-carbinol (I3C), a naturally occurring compound in cruciferous vegetables, is also a direct product of gut microbiota-mediated tryptophan metabolism (45, 46). Previous studies have reported that I3C exhibits anti-inflammatory effects in models of liver disease and general inflammation (45, 47–49). In AD-related research, *Bifidobacterium longum* CCFM1029 was found to produce I3C, which influences Th2 polarisation through the AhR pathway. Animal experiments demonstrated that oral administration of this strain increased faecal and serum I3C levels, accompanied by activation of cutaneous AhR signalling and reductions in inflammatory markers such as TSLP, IL-4, and IL-5. The AhR antagonist CH223191 was able to attenuate these effects, and exogenous I3C could partially mimic them, suggesting that the I3C–AhR axis may be involved in the modulation of AD-like inflammation by this strain. In a corresponding clinical intervention study, after eight weeks of treatment, 70.5% of participants (31/44) were classified as responders (defined by a decrease in SCORAD), who also exhibited increased serum I3C levels that were negatively correlated with SCORAD (*r = –0.4652, p < 0.05*); non-responders showed no significant changes in serum I3C (50). This finding suggests that serum I3C may be associated with treatment response and has potential value as a candidate biomarker. However, given that current evidence is limited to small sample sizes and the causal relationship between responders and non-responders remains unclear, whether serum I3C can serve as a reliable predictor of therapeutic efficacy requires further validation in larger and independent cohorts.

Beyond AhR-related pathways, some studies have focused on metabolite-mediated epigenetic regulation. Alpha-ketoglutarate (α-ketoglutarate, AKG), a key intermediate of the tricarboxylic acid (TCA) cycle, is also a cofactor for dioxygenases and is involved in DNA modification regulation (51–53). Studies have indicated that DNA demethylation can remodel the epigenetic status of *FOXP3* to promote Treg differentiation, whilst histone methylation modifications (such as H3K9me3/H3K27me3) play significant roles in inflammation regulation (54–56). This mechanism provides an additional explanation for the effects of metabolites on immune cell differentiation. Nevertheless, evidence for AKG in AD is primarily from preclinical studies, and the specific epigenetic modifications at key gene loci such as *FOXP3* remain to be fully elucidated.

In summary, molecular interventions in MDTs have provided mechanistic insights into the links between the ‘gut microbiota–metabolite–immune host–skin barrier’ axis. At present, these findings are best considered as candidate mechanisms and potential intervention directions with translational promise.

## 5 Microbial ecological regulation

Although AD is an inflammatory disease, cutaneous microbial dysbiosis is also a significant pathological feature, with increased colonisation by S. aureus being most characteristic and positively correlated with disease severity (57). Consequently, some MDTs focus not only on suppressing the overgrowth of S. aureus, but are also increasingly directed at supplementing or enhancing potentially protective commensal bacteria to restore the cutaneous microbial ecosystem in AD. This section primarily discusses therapeutic strategies that modulate skin microbial colonisation, impacting pathogenic bacterial burden, commensal composition, and local immune-inflammatory responses.

Studies have shown that targeting S. aureus alone provides limited clinical benefit for non-infectious AD patients (58), suggesting that increased S. aureus abundance may be more reflective of microbial imbalance and barrier dysfunction, rather than serving as a sole or direct therapeutic target. Therefore, interventions are gradually shifting from simply inhibiting S. aureus to selecting commensal strains with immunomodulatory properties. For instance, *Staphylococcus cohnii* 2A1(*S. cohnii* 2A1), derived from healthy skin, does not directly inhibit S. aureus growth *in vitro*, but mono-colonisation in germ-free mice alleviates skin inflammation, indicating it may modulate AD-like inflammation through mechanisms beyond direct antimicrobial activity (59). In Th2-type dermatitis models, *S. cohnii* 2A1 downregulated TSLP and Th2-related cytokines (IL-4, IL-13), without significantly increasing Foxp3^+^ regulatory T cells, suggesting a predominant effect on suppressing local Th2 inflammation rather than inducing Treg-mediated immune tolerance. Animal studies also showed symptom improvement within one week and maintenance of anti-inflammatory effects for three weeks without evident attenuation. Further research indicated this strain may modulate the cutaneous inflammatory milieu by promoting local glucocorticoid synthesis and activating anti-inflammatory genes such as *TNFAIP3* and *ATF3 (*59). However, these mechanistic insights are primarily from animal models, and whether they explain the steroid-sparing effect observed in clinical MDT studies requires further validation (60, 61).

Compared to mono-strain colonisation, Myles and colleagues were the first to apply the concept of topical microbiota transplantation in a human trial (62). After 16 weeks of treatment, the paediatric cohort *(n = 5*) showed a significant reduction in the *S. aureus*/*coagulase-negative staphylococci* (*CoNS*) ratio (*P < 0.05*). In the overall cohort (*n = 15*), the SCORAD-50 response rate (>50% improvement) was 66.7% (10/15) and SCORAD-75 response rate was 40% (6/15), with clinical improvement persisting for at least four weeks—substantially exceeding the placebo effect (62).Importantly, no adverse events were reported in either adult or paediatric groups. Additionally, non-responders were associated with a persistent family history of AD into adulthood, suggesting that host genetic background may influence response to microbiota therapies, although this remains an exploratory finding.

To further evaluate the safety and anti-S. aureus efficacy of commensal-based interventions, a phase I randomised double-blind trial (NCT03151148) assessed the effects of *Staphylococcus hominis* A9 (ShA9) in AD patients (63). Mechanistic studies in animal models showed that ShA9 reduced mRNA expression of IL-4, IL-13, and IL-17a and decreased Th2/Th17 infiltration in S. aureus-colonised mice. Clinically, ShA9 rapidly expanded within one hour and reduced S. aureus burden on AD skin. By increasing colonisation with *Staphylococcus epidermidis*, a moderate inhibitory effect on S. aureus was observed within one week. Notably, in some patients, ShA9 did not completely eradicate antibiotic-resistant S. aureus strains but suppressed their virulence gene expression. While no significant improvement in Eczema Area and Severity Index (EASI) was observed in the overall analysis (*P = 0.46*), possibly due to rapid decline of viable bacteria at inflamed sites, safety was favourable (adverse event rate 55.6% vs 83.3%, *P = 0.044*), though the study duration was only one week and long-term efficacy was not assessed (63).

These clinical studies demonstrate the feasibility of modulating the cutaneous microbiota for AD, yet the inflammatory microenvironment and pathogen competition in lesional skin may still limit the delivery, survival, and colonisation of exogenous strains, presenting a key barrier to therapeutic durability. To address this, Liu et al. developed a living bacterial dressing loaded with *Roseomonas mucosa* (Hy@Rm), designed to enhance survival of *R. mucosa* in the AD skin microenvironment and improve competition with *S. aureus* (64). Both *in vitro* and *in vivo* studies showed that this composite material provided protection for *R. mucosa*: compared with free bacteria, the bacterial load in the dressing was significantly higher (approximately four orders of magnitude *in vitro* and 200-fold after 48 hours *in vivo*); Hy@Rm also facilitated *R. mucosa* penetration and colonisation of the epidermis. In animal models, after six weeks of treatment, robust *R. mucosa* colonisation was detected alongside suppression of *S. aureus* proliferation. Ecological modulation with Hy@Rm was associated with improved skin barrier function: at day 14, TEWL and skin hydration were significantly improved. After six weeks of treatment, the animal model showed that the thickness of the mouse epidermis had nearly returned to normal levels, and the mouse dermatitis score was approximately 3, which was markedly lower than that of the free-bacteria group (approximately 7) (64).

This material provides a strain delivery platform for ecological modulation strategies, markedly enhancing the persistence of colonisation in AD skin and demonstrating translational potential. Key unresolved issues include the long-term survival of *R. mucosa* post-treatment, mechanisms of sustained efficacy, and the long-term biocompatibility and immunogenicity of the material’s degradation products.

**Figure 1:**
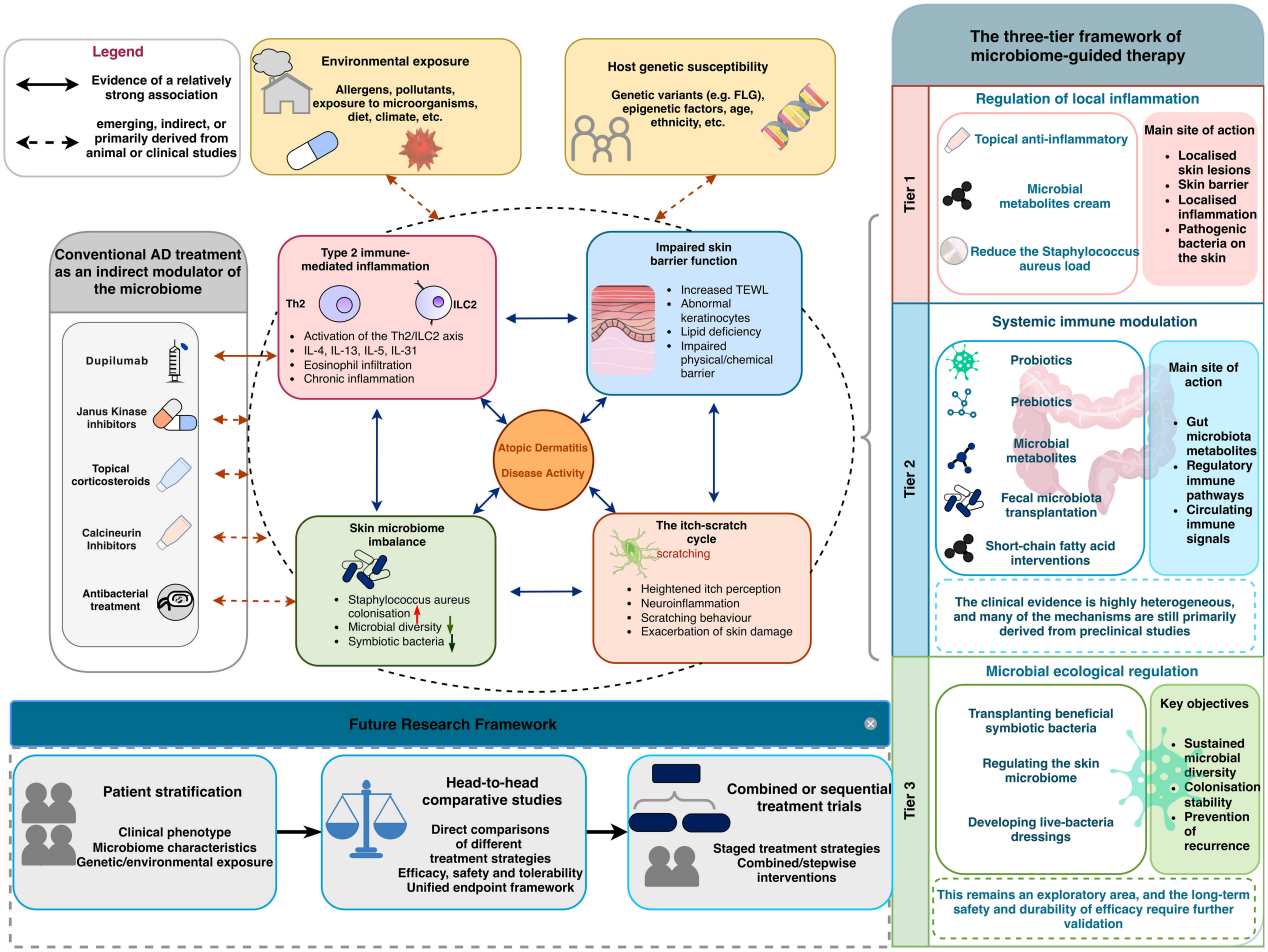
A conceptual model of microbiome-guided therapy for atopic dermatitis. Atopic dermatitis is presented as a multifactorial disease network in which there are bidirectional interactions between epidermal barrier dysfunction, type 2 immune inflammation, skin microbiome dysbiosis and the itch–scratch cycle, and which is jointly influenced by host genetic susceptibility, environmental exposures and gut-derived immunometabolic signals. Microbiome-guided therapy is organised into three non-mutually exclusive tiers: local inflammation control, systemic immunomodulation via the gut–skin axis, and microbiome restoration. Conventional AD treatments may also indirectly reshape the skin microbiome by improving inflammation and barrier function. Solid arrows indicate relationships supported by relatively robust evidence, whilst dotted arrows indicate emerging, indirect relationships or those derived primarily from preclinical studies. Future research should incorporate patient stratification, comparative studies, sequential or combination treatment designs, and long-term follow-up assessments. Image(s) provided by Servier Medical Art (https://smart.servier.com), licensed under CC BY 4.0 (https://creativecommons.org/licenses/by/4.0/).

It should be noted that although some microbial interventions have improved inflammatory or barrier-related indices, there is still a lack of direct evidence that the resultant low-inflammatory state can stably support subsequent combined or sequential microbiota therapies. Therefore, the integration of microbial interventions into long-term AD management remains best considered as a framework for future validation. Based on current research, a three-stage sequential intervention model may be proposed: the first stage focuses on acute local inflammation control to reduce pathogen burden and inflammatory activity; the second stage may explore combination with immunomodulatory therapy to improve host immune status; and the third stage emphasises maintenance of barrier and microbiota stability as a potential adjunct for long-term management (**Figure 1**).

Mechanistically, this framework has some theoretical basis. Firstly, reducing *S. aureus*-associated inflammatory burden may help decrease infection-related exacerbations or treatment interruptions (62, 63); Secondly, microbial interventions may stabilise the local immune microenvironment via modulation of inflammatory gene expression, potentially supporting the use of biologics or Janus kinase (JAK) inhibitors. However, whether this approach can reduce drug dosages or improve long-term response remains to be clinically validated. Similar combination strategies have been explored in other diseases, such as the superior efficacy and safety of rifaximin combined with probiotics versus monotherapy in irritable bowel syndrome (IBS) (65). Thirdly, if microbial interventions can reduce TEWL and improve barrier function, they may theoretically stabilise the local treatment environment and even affect topical drug delivery and utilisation.

Overall, this level of MDTs primarily focuses on cutaneous ecological modulation, aiming to reduce pathogenic burden, promote colonisation by protective commensals, and improve local inflammation and barrier function as adjuncts in AD therapy. Current preclinical and early clinical trials indicate feasibility and safety, but challenges remain for clinical translation, including insufficient persistence of exogenous strain colonisation, unclear long-term safety, substantial patient-to-patient variability in baseline microbiota, the need for optimised delivery systems, and lack of consensus on strain selection and quality control standards. Thus, ecological modulation strategies are best regarded as candidate adjuncts for long-term AD management, with their roles in inflammation control, barrier maintenance, and combined/sequential therapy requiring further clinical validation.

## 6 Conventional AD therapies as indirect microbiome modulators

An important consideration is that, although conventional therapies are not classified as MDTs, changes in the cutaneous microbiome are not exclusive to MDTs. Traditional anti-inflammatory and antimicrobial treatments can also indirectly remodel the microbial community by improving inflammation, barrier integrity, and the local skin microenvironment.

Existing studies have shown that dupilumab can significantly reduce the abundance of *S. aureus* in AD lesions, restore microbial diversity, and increase the number of commensal bacteria (66). Tralokinumab has demonstrated similar microbiome-improving effects to dupilumab (67). Anti-inflammatory treatments, including JAK inhibitors and biologics such as dupilumab, exert their effects by suppressing type 2 inflammation, thereby indirectly altering the microenvironment of lesional skin. Likewise, topical corticosteroids(TCS) can, through their anti-inflammatory and barrier-restorative actions, lead to significant remodelling and normalisation of the density, diversity, and community structure of the cutaneous microbiome in AD lesions (68). Therefore, the improvements in microbial diversity or reduction in S. aureus observed after conventional therapies may be interpreted as both a contributing factor to clinical improvement and a consequence of reduced inflammation and restored barrier function.

Overall, these findings complicate any simplistic causal explanation of dysbiosis in AD. Cutaneous microbial dysbiosis may be both a driver of inflammation and a consequence of barrier disruption and type 2 immune activation. Accordingly, future multidisciplinary therapeutic studies should account for background anti-inflammatory treatments and assess whether microbiota changes constitute a primary mechanism of therapy or are instead a secondary marker of disease control.

## 7 The evidence required for stratified MDTs to progress from proof of concept to clinical validation

The effectiveness of stratified MDTs should not be defined solely by the short-term efficacy of a single intervention compared to placebo. Instead, it should encompass several key aspects:

1) Head-to-head comparative studies. There is a need for direct comparisons of different MDT categories in similar patient populations, under unified endpoint frameworks. Such studies can clarify efficacy, safety, and reproducibility, thereby identifying the optimal therapeutic options for distinct biological subtypes. 2) Sequential therapy studies. It is necessary to verify whether stepwise or escalation treatment strategies are superior to fixed single regimens. Additionally, these studies should assess the impact on overall efficacy and risk exposure. 3) Accompanying biomarker research. Studies should integrate data on the microbiota, metabolites, and immune phenotypes. The aim is to establish multidimensional biomarker systems for patient selection, prediction of therapeutic response, monitoring, and mechanistic explanation. 4) Long-term follow-up studies. These should focus on evaluating the durability of efficacy, relapse rates, colonisation stability of exogenous strains or functional networks, and potential late-onset safety risks.

This core intervention logic is grounded in an overarching methodological framework (**Figure 1**), which ensures the clinical translatability of the model. In other words, the key question for stratified MDTs is not merely ‘Is a given microbiota therapy effective?’, but rather ‘Does an integrated treatment strategy—based on patient stratification and dynamic escalation—outperform traditional, non-stratified empirical therapies?’.

## 8 Summary

The three-tiered framework proposed in this article provides a clear conceptual structure for understanding the heterogeneity of microbiota-directed therapies in atopic dermatitis. The first tier focuses on local inflammation control within skin lesions. The second tier addresses the potential impact of the gut microbiota and its metabolites on systemic immune regulation. The third tier concerns longer-term restoration of the microbial ecosystem. The main contribution of this framework lies in its avoidance of grouping mechanistically distinct interventions—such as probiotics, topical microbial therapies, antimicrobial strategies, and faecal microbiota transplantation—into a single category. Instead, it distinguishes them according to their primary level of action. This distinction helps to explain inconsistencies in existing research findings and provides a theoretical basis for designing future combined or sequential therapeutic strategies.

Nevertheless, there remain clear limitations in the current evidence regarding microbiota-directed therapies for AD. Many mechanistic insights are derived primarily from animal models or *in vitro* experiments, which cannot be directly extrapolated to human disease. Existing clinical studies are also limited by small sample sizes, heterogeneity in intervention strains and dosages, differences in patient age and disease phenotype, and a lack of standardised outcome measures. Furthermore, changes in the cutaneous microbiome can also be observed during conventional treatments, complicating any simplistic causal interpretation of dysbiosis in AD. Cutaneous microbial dysbiosis may act both as a driver of inflammation and as a consequence of barrier disruption and type 2 inflammation. Therefore, therapies targeting the microbiome should be evaluated not only as independent interventions, but also in relation to traditional anti-inflammatory treatments that indirectly remodel the microbial ecosystem.

Future research should clarify which patients, at which disease stages, are most suitable for specific microbiota-directed interventions. Optimal study designs should combine clinical scoring, skin and gut microbiome analysis, metabolomics, immune phenotyping, and assessment of barrier function, with long-term follow-up. Such designs are needed to determine the durability of efficacy, the stability of colonisation by exogenous strains or functional networks, and the presence of delayed safety risks. There is also a need for systematic evaluation of the indirect effects of conventional treatments—such as dupilumab, JAK inhibitors, topical corticosteroids, and topical antimicrobials—on the skin microbiome. In summary, the value of the three-tiered framework lies not in providing immediate new clinical recommendations, but in offering a more systematic theoretical basis for interpreting existing evidence, guiding future trial design, and informing precision microbiota-directed therapeutic strategies.
